# Biotribology in Knee Arthroplasty

**DOI:** 10.1155/2015/618974

**Published:** 2015-03-26

**Authors:** Thomas M. Grupp, Sandra Utzschneider, Markus A. Wimmer

**Affiliations:** ^1^Aesculap AG Research and Development, 78532 Tuttlingen, Germany; ^2^Department of Orthopaedic Surgery, University Hospital of Munich (LMU), Campus Grosshadern, 81377 Munich, Germany; ^3^Section of Tribology, Rush University Medical Center, Chicago, IL 60612, USA

During the last decade, new bearing materials, new methods in in vitro wear simulation, specific cell culture, and animal models to evaluate the response to particulate debris, and dedicated retrieval analysis programs to learn more about material degradation in vivo have been developed in the field of biotribology.

Improvements in knee arthroplasty design, materials, sterilisation techniques, oxidation resistance, and articulating surface treatments have led to superior performance of total knee prostheses by reducing the prevalence of disastrous wear, delamination, and structural fatigue and are expected to show substantial benefits in decreasing wear and osteolysis in the future.

As total knee arthroplasty today is being increasingly performed on younger, heavier, and more active patients, it appears desirable to reduce wear and further improve bearing materials and implant designs in the next decade.

This special issue presents 9 articles with original research papers, a clinical study, and a review article showing different dimensions of biotribology in knee arthroplasty.

The review of A. J. Mitchelson et al. summarizes the available literature in regard to biomaterial hypersensitivity after total knee arthroplasty (TKA) giving supportive evidence and approach consideration for metal ion allergic patients. In this field of knee implant allergy and metal ion hypersensitivity, the research paper of P. Thomas et al. describes a new approach to identify suspected allergy to TKA by combining allergy diagnostics with histopathology and periprosthetic cytokine assessment.

The paper of J. Friesenbichler et al. evaluates the serum ion concentrations of cobalt, chromium, and molybdenum of paediatric tumour patients after fixed hinge knee megaprosthesis in comparison to rotating hinge knee devices and metal-on-metal total hip arthroplasty.

J. Reinders et al. provide a new method of knee wear simulation in vitro based on two different constrained conditions, representing a ligamentous-stable TKA (sacrificed ACL) versus a ligamentous-unstable situation (insufficient anterior and posterior cruciates and medial collateral ligament). V. Ngai and M. A. Wimmer performed gait analysis on thirty TKA patients using the point cluster technique, characterizing a low and a high anterior-posterior motion category as variable inputs for knee wear simulation. A. C. Paulus et al. conducted unicompartmental knee wear testing under bone cement third-body contamination and examined the possible influence on particle size, morphology, and elevated particle numbers.

The study of S. M. Kurtz et al. supports the utility of using multidirectional pin-on-disc screening as a method evaluating wear properties of retrieved polyethylene implant components.

By A. Giurea et al., a clinical study on prospective early results of a modular rotating hinge knee design with carbon-fiber reinforced poly-ether-ether-ketone as a new bearing articulation in TKA was presented, demonstrating no premature material failure or unusual biologic response. Furthermore, within this paper a new classification of failure modes for revision knee arthroplasty was introduced.

